# Food cue-induced craving in individuals with bulimia nervosa and binge-eating disorder

**DOI:** 10.1371/journal.pone.0204151

**Published:** 2018-09-13

**Authors:** Adrian Meule, Carolyn Küppers, Louisa Harms, Hans-Christoph Friederich, Ulrike Schmidt, Jens Blechert, Timo Brockmeyer

**Affiliations:** 1 Department of Psychology, University of Salzburg, Salzburg, Austria; 2 Centre for Cognitive Neuroscience, University of Salzburg, Salzburg, Austria; 3 Department of General Internal Medicine and Psychosomatics, Center for Psychosocial Medicine, Heidelberg University Hospital, Heidelberg, Germany; 4 Department of Psychosomatic Medicine and Psychotherapy, Medical Faculty, Heinrich-Heine-University Düsseldorf, Düsseldorf, Germany; 5 Section of Eating Disorders, Department of Psychological Medicine, Institute of Psychiatry, Psychology and Neuroscience, King’s College London, London, United Kingdom; 6 Eating Disorders Unit, South London and Maudsley NHS Foundation Trust, London, United Kingdom; 7 Department of Clinical Psychology and Psychotherapy, Institute of Psychology, University of Goettingen, Goettingen, Germany; Universitat de Barcelona, SPAIN

## Abstract

Individuals with bulimia nervosa (BN) or binge-eating disorder (BED) experience more frequent and intense food cravings than individuals without binge eating. However, it is currently unclear whether they also show larger food cue-induced increases in craving (i.e., food cue reactivity) than those without binge eating, as suggested by conditioning theories of binge eating. A group of individuals with BN or BED (binge-eating group, *n* = 27) and a group of individuals with low trait food craving scores and without binge eating (control group, *n* = 19) reported their current food craving before and after a food cue exposure. Although food craving intensity significantly increased in both groups, this increase was significantly stronger in the binge-eating group than in the control group. This result is in line with conditioning models of binge eating that propose that food cues are conditioned stimuli that elicit a conditioned response (e.g., food craving) and that this association is stronger in individuals with binge eating. As food craving increased in individuals with low trait food craving scores as well—although to a lesser extent—previous null results might be explained by methodological considerations such as not screening control participants for trait food craving.

## Introduction

In terms of classical conditioning, food intake may be considered an unconditioned stimulus and its metabolic effects unconditioned responses. Cues that reliably signal food intake (e.g., the sight, smell, and taste of food) may start to act as conditioned stimuli that can trigger conditioned responses. This learned food cue reactivity can manifest in several objectively-measurable physiological responses (e.g., increases in salivary flow, heart rate) upon exposure to food or food cues, yet an essential element of food cue reactivity is a subjective experience of an intense desire to eat the food (i.e., craving) [1].

The conditioning model of binge eating proposes that individuals with binge eating display higher reactivity to food cues than individuals without binge eating, which ultimately leads to excessive food intake [2]. However, when examining studies that measured self-reported food craving, it appears that the assumption of larger food cue-induced increases in craving in individuals with binge eating (e.g., persons with bulimia nervosa [BN] or binge-eating disorder [BED]) has not been reliably established.

First, individuals with binge eating do indeed report higher trait food craving (i.e., experience more frequent and intense food cravings in general) and higher state food craving (i.e., experience more intense food craving in the moment of data collection) than individuals without binge eating [3, 4]. However, such studies cannot answer the question of whether individuals with BN or BED show higher food cue *reactivity*—in terms of food cue-induced increases in craving—than healthy controls. Second, some studies reported higher food craving in individuals with BN or BED than in healthy controls after a food cue exposure, but did not measure baseline levels of food craving [5, 6]. Thus, whether craving increases were larger in participants with BN or BED than in controls cannot be inferred from these studies. Third, studies that included a food cue exposure with pre- and post-exposure measurements of food craving and compared individuals with BN and/or BED with healthy controls did not find a significant interaction between groups and measurements, that is, food craving or desire to binge increased equally in all groups during food cue exposure [7–14].

In conclusion, while individuals with BN or BED report more frequent and intense food cravings in general than individuals without binge eating, there is no compelling evidence that these differences reflect an elevated reactivity to food cues in terms of larger increases of food craving intensity in response to food cues (which would explain their difficulties in controlling food intake). Therefore, we tested whether a group of individuals with BN or BED would demonstrate stronger increases in momentary food craving during a food cue exposure than individuals without binge eating. Previous null findings may be partially due to the fact that control participants were not screened for trait food craving levels, which would also be associated with elevated food cue reactivity [15, 16]. Thus, to ensure that control participants did not have any sub-threshold eating disorder symptoms or any eating-related psychopathology, we used a control group of individuals without binge eating and low levels of trait food craving.

## Materials and methods

### Participants

The study was approved by the institutional review board of the Medical Faculty of the University of Heidelberg. Participants provided written informed consent before commencing the study and received financial compensation for their participation in the study. Fourteen individuals with BN and 13 individuals with BED, who took part in a larger research project [17] and who were all recruited at the same study site, participated in this study (binge-eating group). Nineteen individuals without any eating disorder or elevated trait food craving scores were used as control group [18]. Sample characteristics are displayed in Table 1. In the binge-eating group, 66.7% of participants had normal weight (*n* = 18), 14.8% were overweight (*n* = 4), and 18.5% were obese (*n* = 5), according to the guidelines by the World Health Organization [19]. In the control group, 78.9% of participants had normal weight (*n* = 15), 15.8% were overweight (*n* = 3), and 5.3% were obese (*n* = 1).

**Table 1:** Sample characteristics as a function of group.

	Binge-eating group (*n* = 27)	Control group (*n* = 19)	Test statistics
Age (years)	*M* = 30.0 (*SD* = 11.5)	*M* = 24.4 (*SD* = 3.20)	*t*_(31.5)_ = 2.42, *p* = .021, *d* = 0.62
Sex (women)	*n* = 24 (88.9%)	*n* = 17 (89.5%)	χ^2^_(1)_ = 0.004, *p* = .950, *d* = 0.02
Education (A-level)	*n* = 26 (96.3%)	*n* = 17 (89.5%)	χ^2^_(1)_ = 0.85, *p* = .356, *d* = 0.27
Body mass index (kg/m^2^)	*M* = 24.2 (*SD* = 5.27)	*M* = 22.6 (*SD* = 3.80)	*t*_(43.9)_ = 1.21, *p* = .233, *d* = 0.34
Eating Disorder Examination–Questionnaire	*M* = 3.01 (*SD* = 0.81)	*M* = 0.87 (*SD* = 0.70)	*t*_(44)_ = 9.30, *p* < .001, *d* = 2.79
Number of binge days in the past 28 days	*M* = 14.7 (*SD* = 7.14)	—	—
Food Cravings Questionnaire–State (pre exposure)	*M* = 40.1 (*SD* = 12.4)	*M* = 34.0 (*SD* = 11.8)	*t*_(44)_ = 1.67, *p* = .102, *d* = 0.50
Food Cravings Questionnaire–State (post exposure)	*M* = 48.6 (*SD* = 13.7)	*M* = 38.7 (*SD* = 11.8)	*t*_(44)_ = 2.54, *p* = .015, *d* = 0.76

All participants were recruited through websites, circular mails, advertising posters and advertisements in local and social media. Women and men were eligible for participation in the study if they were aged 18 years or above and had a body mass index ≥ 18.5 kg/m^2^. Diagnostic assessment in the binge-eating group was based on the *Structured Clinical Interview for DSM-5 Disorders* [20]. Exclusion criteria for the binge-eating group were: medical (e.g., severe electrolyte abnormalities) or psychiatric (e.g., acute suicidality) instability, the need for immediate inpatient treatment, substance dependence, psychosis, bipolar disorder, borderline personality disorder, psychotropic medication use, severe learning disability or inability to speak fluent German, impacting on the person’s ability to complete study assessments. Use of selective serotonin reuptake inhibitors was not an exclusion criterion when medication was stable (i.e. at least 14 days of continuous use). Participants in the control group were invited for laboratory testing if they scored in the lower third on the German version of the *Food Cravings Questionnaire–Trait* [21] in a sample of *n* = 358 volunteers, did not report any binge eating episodes as assessed with the *Eating Disorder Examination–Questionnaire* (EDE–Q) [22] and had no current or prior psychotherapeutic or psychopharmacological treatment.

### Measures

#### Food cue exposure

A five-minute video clip was used to induce food craving. The video contains clips from television advertisements including palatable (both junk and non-junk) foods. The video has been previously used by Kekic and colleagues who reported that the foods shown were rated as highly appetizing and that hunger was significantly increased after watching the video [23].

#### Food Cravings Questionnaire–State (FCQ–S)

The German version of the FCQ–S [21] was used for measuring momentary food craving intensity. The scale has 15 items (e.g., “I have an intense desire to eat [one or more specific foods].”). Participants are asked to indicate on a five-point scale the extent to which they agree with each statement *right now*, *at this very moment*, ranging from *strongly disagree* to *strongly agree* [24]. Thus, total scores can range between 15 and 75. Cepeda-Benito and colleagues [24] originally proposed a five-factor model of the FCQ–S. However, as the scale usually has very high internal reliability and as studies that examined its factor structure have been inconsistent [9, 21, 25–27], only the total score was used in the analyses. Internal reliability was high both before (α = .923) and after (α = .943) food cue exposure.

### Procedure

Participants were asked to refrain from eating food, drinking caffeine-containing beverages, and smoking in the two hours before the assessment. They were tested in the laboratory individually and completed the FCQ–S. Following this, the five-minute video clip was shown as food cue exposure. Subsequently, participants completed the FCQ–S again. Finally, body height and weight were measured.

### Data analyses

Differences between groups were tested with independent samples *t*-tests (age, body mass index, EDE–Q scores) and χ^2^-tests (sex, education). Regarding state food craving, an analysis of variance for repeated measures was calculated with *group* (binge eating vs. control) as between-subjects factor, *measurement* (before vs. after food cue exposure) as within-subjects factor, and FCQ–S scores as dependent variable. All data analyses were conducted with IBM SPSS Statistics Version 20.

## Results

**Fig 1:**
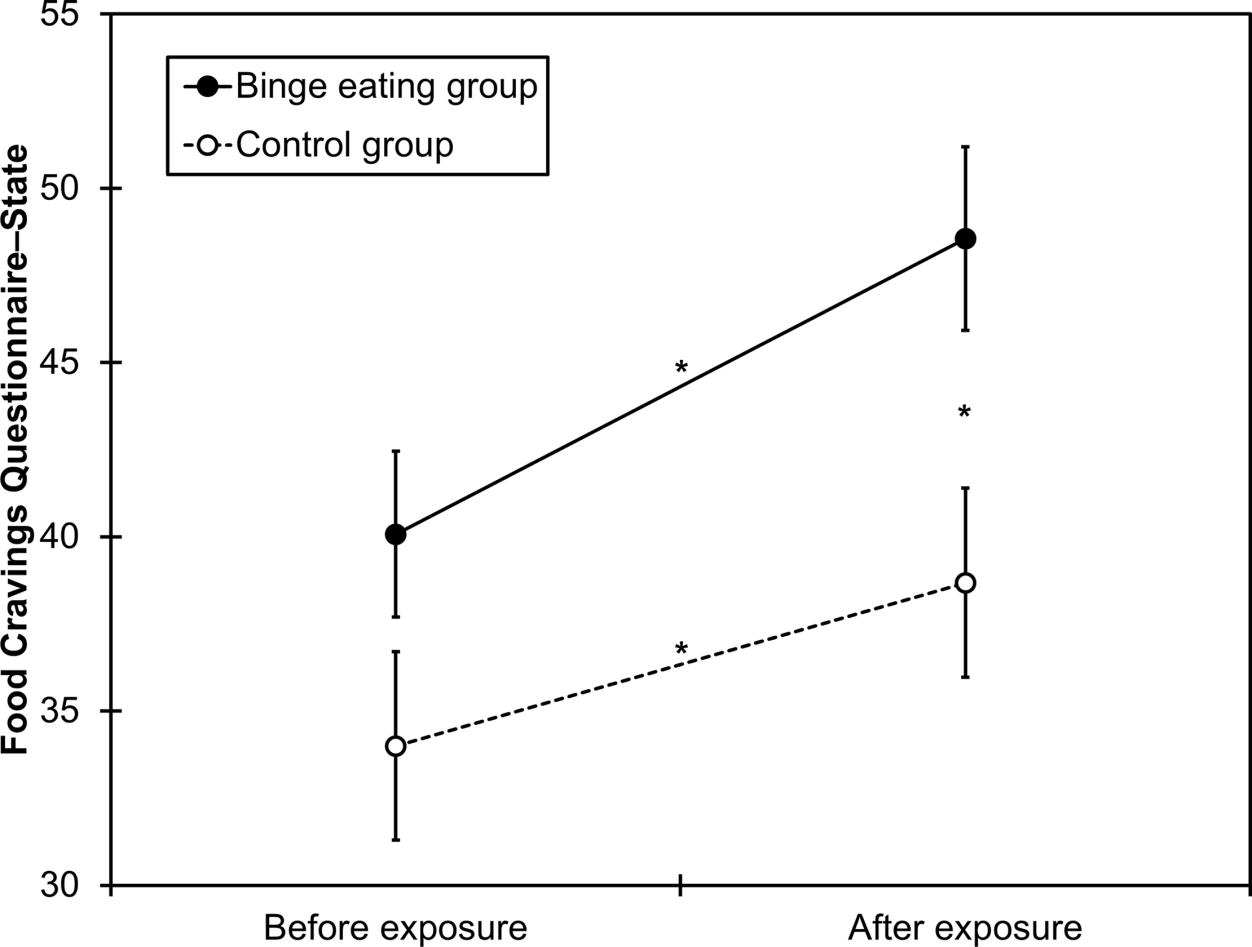
Scores on the Food Cravings Questionnaire–State as a function of group (controls vs. individuals with bulimia nervosa or binge-eating disorder) before and after food cue exposure. Error bars represent the standard error of the mean. Asterisks indicate *p* < .05.

The binge-eating group was older and had higher EDE–Q scores than the control group (Table 1). Regarding state food craving, main effects of *group* (*F*_(1,44)_ = 4.78, *p* = .034, η_p_^2^ = .098) and *measurement* (*F*_(1,44)_ = 51.6, *p* < .001, η_p_^2^ = .540) were qualified by a significant *group* × *measurement* interaction (*F*_(1,44)_ = 4.29, *p* = .044, η_p_^2^ = .089). During the food cue exposure, state food craving increased in both the binge-eating group (*t*_(26)_ = 6.58, *p* < .001, *d* = 0.64) and the control group (*t*_(18)_ = 3.95, *p* = .001, *d* = 0.40). However, while groups did not differ before the food cue exposure, the binge-eating group had higher state food craving than the control group after the food cue exposure (Table 1; Fig 1).

Note that the age difference between groups was driven by participants with BED (*M* = 35.2 years, *SD* = 13.5) who were older than participants with BN (*M* = 25.2 years, *SD* = 6.62, *t*_(17.2)_ = 2.41, *p* = .028, *d* = 0.95). As individuals with BED are commonly older than individuals with BN on average, it would have been inappropriate to use age as covariate in the analysis of variance [28]. Thus, we examined effects after excluding the three oldest participants (>50 years old) such that the binge eating and control group did not differ in age any more (*t*_(31.8)_ = 1.49, *p* = .147, *d* = 0.42). Using these age-matched groups, the *group* × *measurement* interaction was still significant (*F*_(1,41)_ = 5.45, *p* = .025, η_p_^2^ = .117).

## Discussion

Numerous studies showed that individuals with BN or BED experience more frequent and more intense food cravings than persons without binge eating [3–6]. Yet, food cue-induced craving did not differ in individuals with and without binge eating in the majority of studies [7–14], raising the question whether cue reactivity is a useful concept to explain binge eating.

In the current study, individuals with and without binge eating did not differ in their momentary food craving prior to food cue exposure. While it has been found that high trait food craving scores (which individuals with BN or BED have) relate to higher state food craving scores even without being exposed to food cues, this association is rather small [29]. Thus, a difference in state food craving between individuals with and without binge eating at baseline may only be detected in larger samples.

During food cue exposure, state food craving intensity significantly increased in both groups in the current study. However, individuals with BN or BED showed significantly larger food cue reactivity in terms of self-reported craving. Of note, the food cue exposure induced craving even in the control group who was explicitly recruited to have low trait food craving scores. Therefore, we speculate that the lack of finding interactive effects of groups and measurements in previous studies may be due to participant selection. For instance, several previous studies only examined analogue samples, participants with sub-threshold BN/BED, or very small samples [7–9, 11, 13]. Moreover, previous study designs may not have been sensitive enough to detect differences in food cue-induced craving because high calorie foods appeal to all humans and, likewise, induce craving in most individuals (as was demonstrated by craving changes in our low trait food craving control group, in line with previous findings [15]). Taken together, this suggests that differential effects of food cue exposure may only be observed when groups are clearly separable; not only with regard to eating disorder pathology but also with regard to other relevant aspects such as trait food craving scores.

Some methodological considerations limit interpretation of the current findings. First, the sample was predominantly composed of non-obese women. Thus, findings may not be applicable to men or individuals with obesity. Second, because of the small number of individuals with BN and BED, they were combined to one binge-eating group. However, some differences between individuals BN and BED have been noted, for example, with regard to binge eating episodes characteristics [30, 31]. Thus, future studies may reveal differences in food cue reactivity between these groups as well. Third, we did not include a control group of high trait food cravers without binge eating or any other eating-related psychopathology. Recruiting such a control group may be hard to achieve because of the large overlap between trait food craving scores and binge eating tendencies [21]. Yet, as elevated food cue reactivity in high trait food cravers has been previously demonstrated [15], we speculate that such a control group would show similar cue-induced increases in food craving to individuals with BN or BED.

## Conclusions

Our data are in line with classical conditioning accounts which propose that food cues represent conditioned stimuli that trigger a conditioned response (e.g., food craving) and that these associations are stronger in individuals with binge eating than in healthy individuals. Although we did not assess actual food intake in the current study, this elevated food cue reactivity may ultimately increase the likelihood to engage in binge eating [2, 32]. As food craving increased in individuals with low trait food craving scores in the current study as well, previous null results might be explained by methodological considerations such as not screening control participants for trait food craving.

## Supporting information

S1 Data Study data. (SAV)

## References

[1] Nederkoorn C, Smulders F, Jansen A. Cephalic phase responses, craving and food intake in normal subjects. Appetite. 2000;35: 45–55. 10.1006/appe.2000.0328 10896760

[2] Jansen A. A learning model of binge eating: cue reactivity and cue exposure. Behav Res Ther. 1998;36: 257–272. 10.1016/S0005-7967(98)00055-2 9642846

[3] Innamorati M, Imperatori C, Balsamo M, Tamburello S, Belvederi Murri M, Contardi A, et al Food Cravings Questionnaire–Trait (FCQ–T) discriminates between obese and overweight patients with and without binge eating tendencies: The Italian Version of the FCQ–T. J Pers Assess. 2014;96: 632–639. 10.1080/00223891.2014.909449 24793741

[4] Moreno S, Rodríguez S, Fernandez MC, Tamez J, Cepeda-Benito A. Clinical validation of the trait and state versions of the Food Craving Questionnaire. Assessment. 2008;15: 375–387. 10.1177/1073191107312651 18310596

[5] Ferrer-Garcia M, Pla-Sanjuanelo J, Dakanalis A, Vilalta-Abella F, Riva G, Fernandez-Aranda F, et al Eating behavior style predicts craving and anxiety experienced in food-related virtual environments by patients with eating disorders and healthy controls. Appetite. 2017;117: 284–293. 10.1016/j.appet.2017.07.007 28709960

[6] Van den Eynde F, Koskina A, Syrad H, Guillaume S, Broadbent H, Campbell IC, et al State and trait food craving in people with bulimic eating disorders. Eat Behav. 2012;13: 414–417. 10.1016/j.eatbeh.2012.07.007 23121801

[7] Geliebter A, Benson L, Pantazatos SP, Hirsch J, Carnell S. Greater anterior cingulate activation and connectivity in response to visual and auditory high-calorie food cues in binge eating: preliminary findings. Appetite. 2016;96: 195–202. 10.1016/j.appet.2015.08.009 26275334 PMC4684801

[8] Legenbauer T, Vögele C, Rüddel H. Anticipatory effects of food exposure in women diagnosed with bulimia nervosa. Appetite. 2004;42: 33–40. 10.1016/S0195-6663(03)00114-4 15036781

[9] Lombardo C, Iani L, Barbaranelli C. Validation of an Italian version of the Food Craving Questionnaire-State: Factor structure and sensitivity to manipulation. Eat Behav. 2016;22: 182–187. 10.1016/j.eatbeh.2016.06.003 27294790

[10] Nederkoorn C, Smulders F, Havermans R, Jansen A. Exposure to binge food in bulimia nervosa: finger pulse amplitude as a potential measure of urge to eat and predictor of food intake. Appetite. 2004;42: 125–130. 10.1016/j.appet.2003.11.001 15010175

[11] Ng L, Davis C. Cravings and food consumption in binge eating disorder. Eat Behav. 2013;14: 472–475. 10.1016/j.eatbeh.2013.08.011 24183139

[12] Sobik L, Hutchison K, Craighead L. Cue-elicited craving for food: a fresh approach to the study of binge eating. Appetite. 2005;44: 253–261. 10.1016/j.appet.2004.12.001 15876472

[13] Vögele C, Florin I. Psychophysiological responses to food exposure: an experimental study in binge eaters. Int J Eat Disord. 1997;21: 147–157. 10.1002/(SICI)1098-108X(199703)21:2<147::AID-EAT5>3.0.CO;2-L 9062838

[14] Wolz I, Sauvaget A, Granero R, Mestre-Bach G, Baño M, Martín-Romera V, et al Subjective craving and event-related brain response to olfactory and visual chocolate cues in binge-eating and healthy individuals. Sci Rep. 2017;7: 1–10. 10.1038/s41598-016-0028-x 28155875 PMC5290481

[15] Meule A, Skirde A, Freund R, Vögele C, Kübler A. High-calorie food-cues impair working memory performance in high and low food cravers. Appetite. 2012;59: 264–269. 10.1016/j.appet.2012.05.010 22613059

[16] Meule A, Hermann T, Kübler A. A short version of the Food Cravings Questionnaire-Trait: The FCQ-T-reduced. Front Psychol. 2014;5: 1–10. 10.3389/fpsyg.2014.00001 24624116 PMC3940888

[17] Brockmeyer T, Schmidt U, Friederich HC. The ABBA study–approach bias modification in bulimia nervosa and binge eating disorder: study protocol for a randomised controlled trial. Trials. 2016;17: 1–9. 10.1186/s13063-015-1128-9 27670138 PMC5037622

[18] Brockmeyer T, Hahn C, Reetz C, Schmidt U, Friederich HC. Approach bias and cue reactivity towards food in people with high versus low levels of food craving. Appetite. 2015;95: 197–202. 10.1016/j.appet.2015.07.013 26184338

[19] World Health Organization. Obesity: preventing and managing the global epidemic Geneva: World Health Organization; 2000. 11234459

[20] First MB, Williams JBW, Karg RS, Spitzer RL. Structured Clinical Interview for DSM-5 Disorders Washington, DC: American Psychiatric Association Publishing; 2016.

[21] Meule A, Lutz A, Vögele C, Kübler A. Food cravings discriminate differentially between successful and unsuccessful dieters and non-dieters. Validation of the Food Cravings Questionnaires in German. Appetite. 2012; 58: 88–97. 10.1016/j.appet.2011.09.010 21983051

[22] Hilbert A, Tuschen-Caffier B. Eating Disorder Examination–Questionnaire Tübingen, Germany: dgvt Verlag; 2016.

[23] Kekic M, McClelland J, Bartholdy S, Boysen E, Musiat P, Dalton B, et al Single-session transcranial direct current stimulation temporarily improves symptoms, mood, and self-regulatory control in bulimia nervosa: a randomised controlled trial. PLoS ONE. 2017;12: 1–17. 10.1371/journal.pone.0167606 28121991 PMC5266208

[24] Cepeda-Benito A, Gleaves DH, Williams TL, Erath SA. The development and validation of the state and trait Food-Cravings Questionnaires. Behav Ther. 2000;31: 151–173. 10.1016/S0005-7894(00)80009-X 11060941

[25] Nijs IMT, Franken IHA, Muris P. The modified Trait and State Food-Cravings Questionnaires: Development and validation of a general index of food craving. Appetite. 2007;49: 38–46. 10.1016/j.appet.2006.11.001 17187897

[26] Vander Wal JS, Johnston KA, Dhurandhar NV. Psychometric properties of the State and Trait Food Cravings Questionnaires among overweight and obese persons. Eat Behav. 2007;8: 211–223. 10.1016/j.eatbeh.2006.06.002 17336791

[27] Queiroz de Medeiros AC, Campos Pedrosa LF, Hutz CS, Yamamoto ME. Brazilian version of food cravings questionnaires: Psychometric properties and sex differences. Appetite. 2016;105: 328–333. 10.1016/j.appet.2016.06.003 27288149

[28] Miller GA, Chapman JP. Misunderstanding analysis of covariance. J Abnorm Psychol. 2001;110: 40–48. 10.1037/0021-843X.110.1.40 11261398

[29] Meule A, Beck Teran C, Berker J, Gründel T, Mayerhofer M, Platte P. On the differentiation between trait and state food craving: Half-year retest-reliability of the Food Cravings Questionnaire-Trait-reduced (FCQ-T-r) and the Food Cravings Questionnaire-State (FCQ-S). J Eat Disord. 2014;2: 1–3. 10.1186/2050-2974-2-1 25356313 PMC4212121

[30] Mitchell JE, Mussell MP, Peterson CB, Crow S, Wonderlich SA, Crosby RD, et al Hedonics of binge eating in women with bulimia nervosa and binge eating disorder. Int J Eat Disord. 1999;26: 165–170. 10.1002/(Sici)1098-108x(199909)26:2<165::Aid-Eat5>3.0.Co;2-H 10422605

[31] Fitzgibbon ML, Blackman LR. Binge eating disorder and bulimia nervosa: Differences in the quality and quantity of binge eating episodes. Int J Eat Disord. 2000;27: 238–243. 10.1002/(Sici)1098-108x(200003)27:2<238::Aid-Eat12>3.0.Co;2-Q 10657897

[32] Waters A, Hill A, Waller G. Bulimics’ responses to food cravings: is binge-eating a product of hunger or emotional state? Behav Res Ther. 2001;39: 877–886. 10.1016/S0005-7967(00)00059-0 11480829

